# Functional analysis of *HECA* variants identified in congenital heart disease in the Chinese population

**DOI:** 10.1002/jcla.24649

**Published:** 2022-08-10

**Authors:** Ting Li, Yao Wu, Wei‐Cheng Chen, Xing Xue, Mei‐Jiao Suo, Ping Li, Wei Sheng, Guo‐Ying Huang

**Affiliations:** ^1^ Children's Hospital of Fudan University Shanghai China; ^2^ Shanghai Key Laboratory of Birth Defects Shanghai China; ^3^ Research Unit of Early Intervention of Genetically Related Childhood Cardiovascular Diseases(2018RU002) Chinese Academy of Medical Sciences Shanghai China

**Keywords:** AC16 cells, association, cell cycle, cell proliferation, congenital heart disease, *HECA*, variation

## Abstract

**Background:**

Congenital heart disease (CHD) is a class of cardiovascular defects that includes septal defects, outflow tract abnormalities, and valve defects. Human homolog of Drosophila headcase (*HECA*) is a novel cell cycle regulator whose role in CHD has not been elucidated. This is the first study to determine the frequency of *HECA* mutations in patients with CHD and the association between *HECA* variants and CHD.

**Methods:**

In this study, we identified a candidate gene, *HECA*, by whole‐exome sequencing of an atrial septal defect family. To investigate the association between *HECA* variants and CHD risk, targeted exon sequencing was conducted in 689 individuals with sporadic CHD. We further analyzed the effect of *HECA* gene abnormalities on cardiomyocyte phenotype behavior and related signaling pathways by Western blotting, reverse transcription‐quantitative polymerase chain reaction, and scratch assay.

**Results:**

We found a novel de novo mutation, c.409_410insA (p. W137fs), in the *HECA* gene and identified five rare deleterious variants that met the filtering criteria in 689 individuals with sporadic CHD. Fisher's exact test revealed a significant association between *HECA* variations and CHD compared with those in gnomADv2‐East Asians(*p* = 0.0027). Further functional analysis suggested that the variant p. W137fs resulted in a deficiency of the normal HECA protein, and HECA deficiency altered AC16 cell cycle progression, increased cell proliferation, and migration, and promoted the activation of the PDGF‐BB/PDGFRB/AKT pathway.

**Conclusions:**

Our study identified *HECA* and its six rare variants, expanding the spectrum of genes associated with CHD pathogenesis in the Chinese population.

## Graphical abstract

In this study, we identified a candidate gene *HECA* and its six rare variants, and Fisher's exact test reveals a significant association of HECA variations with CHD, comparing with those in gnomADv2‐East Asian. Further functional analysis suggested that the variant *p*.W137fs resulted in the deficiency of the normal HECA protein, and HECA deficiency altered AC16 cell cycle progression, increase cell proliferation and migration, and promoted activation of the PDGF‐BB/PDGFRB/AKT pathway.

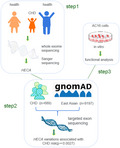

## 1 INTRODUCTION

Congenital heart disease (CHD) is a malformation of the heart or large blood vessels with abnormal embryonic development and is the most common birth defect.^1^ From 1970 to 2017, the global prevalence of CHD gradually increased.^2^ A prospective study reported that the overall prevalence of CHD in China was 8.98/1000.^3^ Epidemiological studies have pointed out that approximately 20–30% of CHD cases are due to environmental or genetic reasons, while other unexplained CHD cases are considered to be the result of multiple factors.^4^, ^5^, ^6^ Around 400 genes have been reported to be closely related to CHD pathogenesis.^7^ These genetic variants are mainly concentrated in transcription factors, cell signaling and adhesion proteins, and structural proteins that are closely involved in cardiovascular development.^8^, ^9^ The development of the heart from progenitor cells into a functionally mature four‐chambered heart requires rapid cardiomyocyte proliferation and increased cardiomyocyte size. Cardiac development is an extremely complex and delicate process, and the precise control of cardiomyocyte proliferation is critical for normal development of the heart. Imbalances in the regulation of cell cycle and cell proliferation during cardiac development can cause abnormal cardiac development and lead to CHD.^10^ For example, *CDC42*,^11^ *CITED2*,^12^ *HAND2*,^13^, ^14^ *MESP2*,^15^ and other factors that regulate cardiomyocyte proliferation have been shown to be involved in cardiac development and CHD.

Human homolog of Drosophila headcase (*HECA*) is a candidate tumor suppressor gene located on chromosome 6q23–24 and is a novel cell cycle regulator.^16^ In Drosophila, headcase regulates cell proliferation and differentiation during adult morphogenesis and may affect the JAK/STAT and Wnt/β‐catenin pathways.^17^, ^18^, ^19^ The human HECA protein is highly similar to the Drosophila headcase, and its effects on human tissues and the corresponding molecular mechanisms may be similar to those in Drosophila.^20^ In humans, HECA is aberrantly expressed in head and neck,^21^ colorectal,^16^ and pancreatic cancers.^22^ Studies on oral squamous cell carcinoma have shown that the HECA promoter is a target of the Wnt/β‐catenin pathway and that HECA protein antagonizes Wnt/β‐catenin pathway‐mediated cell proliferation.^21^

Based on our knowledge of gene function, we speculated that the abnormal expression and function of the HECA protein may play a role in cardiac development and the pathogenesis of CHD by affecting the proliferation of cardiomyocytes. To date, there have been no reports of *HECA* in patients with CHD, and research on *HECA* mainly focused on tumors. To confirm the role of *HECA* in CHD, we performed whole‐exome sequencing (WES) in a family with atrial septal defect (ASD) and 689 patients with sporadic CHD, and analyzed the association of rare variants of *HECA* with CHD risk. We then overexpressed the p. W137fs mutation and downregulated the HECA protein in cardiomyocytes to determine the effect of abnormal HECA on cardiomyocyte phenotypic behavior and the corresponding molecular mechanisms.

## 2 MATERIALS AND METHODS

### 2.1 Study subjects

From May 2015 to December 2020, we recruited a pedigree of ASD and 689 patients with sporadic CHD at the Children's Hospital of Fudan University. Among those with sporadic CHD, cases with chromosomal abnormalities and syndromes were excluded. The clinical characteristics of the 689 sporadic CHD cases are summarized in Table 1. All participants or their guardians provided written informed consent in accordance with the Declaration of Helsinki. Our research was approved by the Ethics Committee of the Children's Hospital of Fudan University (code number: 2016121).

**TABLE 1:** Clinical characteristics of patients with sporadic CHD

Characteristic	NO.	Gender		Age (months) Median (IQR)
		Male (%)	Female (%)	
Lesions causing outflow obstruction				
CoA	6	3 (50%)	3 (50%)	5.08 (2.28–6.44)
AS	6	2 (33.33%)	4 (66.67%)	71.57 (16.67–111.59)
IAA	1	1 (100%)	0	3.33
PS	4	3 (75%)	1 (25%)	10.32 (1.18–33.63)
Lesions causing left‐to‐right shunting				
VSD	197	127 (64.47%)	70 (35.53%)	5.23 (2.97–13.27)
PDA	13	4 (30.77%)	9 (69.23%)	6.40 (4.83–12.30)
ASD	78	45 (57.69%)	33 (42.31%)	11.45 (5.77–26.25)
AVSD	20	10 (50%)	10 (50%)	13.10 (5.52–46.28)
PAPVR	5	3 (60%)	2 (40%)	18.57 (18.03–21.33)
Lesions causing right‐to‐left shunting				
TOF	147	88 (59.86%)	59 (40.14%)	7.97 (5.78–16.93)
PA	7	5 (71.43%)	2 (28.57%)	2.87 (1.12–144.70)
PA‐VSD	26	15 (57.69%)	11 (42.31%)	9.13 (1.19–60.03)
Ebstein	3	3 (100%)	0	4.10 (2.07–17.87)
TGA	80	58 (72.50%)	22 (27.50%)	0.87 (0.17–5.66)
SV	7	6 (85.71%)	1 (14.29%)	55.53 (46.12–67.93)
DORV	58	39 (67.24%)	19 (32.76%)	9.52 (3.63–45.63)
TAPVR	8	5 (62.50%)	3 (37.50%)	2.97 (1.30–5.88)
others	23	13 (56.52%)	10 (43.48%)	62.53 (6.85–81.48)
Total	689	430 (62.41%)	259 (37.59%)	7.23 (3.03–21.33)

Abbreviations: AS, aortic stenosis; ASD, atrial septal defect; AVSD, atrioventricular septal defect; CoA, coarctation of the aorta; DORV, double‐outlet right ventricle; Ebstein, Ebstein's anomaly; IAA, interruption of aortic arch; PA, pulmonary atresia; PAPVR, partial anomalous pulmonary venous return; PA‐VSD, pulmonary atresia with ventricular septal defect; PDA, patent ductus arteriosus; PS, pulmonic valve stenosis; SV, single ventricle; TAPVR, total anomalous pulmonary venous return; TGA, transposition of the great arteries; TOF, tetralogy of Fallot; VSD, ventricular septal defect.

### 2.2 Whole‐exome sequencing and Sanger sequencing

Genomic DNA samples were obtained from the peripheral venous blood of family members and all sporadic patients using the QIAamp DNA Blood Mini Kit (Qiagen) according to the manufacturer's specifications. Whole‐exome sequencing (WES) was performed by Gemple Biotech Co., Ltd. Population allele frequencies were obtained from GnomADv2 (Genome Aggregation Database, https://gnomad.broadinstitute.org/) and predicted to be deleterious by CADD (combined annotation dependent depletion, https://cadd.gs.washington.edu/snv), SIFT (sorting intolerant from tolerant, https://sift.bii.a‐star.edu.sg/), PolyPhen2 (prediction of functional effects of human nsSNPs, http://genetics.bwh.harvard.edu/pph2/), and MutationTaster (https://www.mutationtaster.org/). We screened for rare and predicted highly deleterious coding variants that met the filtering criteria (absence or gnomADv2_exome_EAS frequency ≤0.01% and CADD score > 20). We then validated the screened rare variants using Sanger sequencing. As listed in Table S1, the sequences of the forward and reverse primers were designed using the NCBI Primer designing tool online (https://www.ncbi.nlm.nih.gov/tools/primer‐blast/). Polymerase chain reaction (PCR) was performed using PrimeSTAR® Max DNA Polymerase according to the manufacturer's specifications. Precisiongenes Technology, Inc., sequenced the PCR products. Mutation Surveyor Software was used to analyze the sequence data.

Reference control data were obtained from GnomADv2, which contains exome sequencing data from 125,748 unrelated individuals. Considering ethnic specificity, we only selected East Asian populations (*n* = 9197) in gnomADv2 as the control group. The filtering criteria for rare pathogenic variants in the reference control population were consistent with those in our patients with CHD

### 2.3 Cell culture and passage

HEK293T and AC16 cells were cultivated in Dulbecco's modified Eagle's medium (1×) (Gibco, USA) supplemented with 10% fetal bovine serum (Gibco, USA) at 37°C and 5% CO_2_. Cells were washed with phosphate‐buffered saline (PBS) (Biosharp, China) and digested with 0.25% trypsin–EDTA (Gibco, USA) for passage.

### 2.4 Plasmid construction and transfection

The pcDNA3.1‐*HECA* WT‐3xMyc and pcDNA3.1‐*HECA* W137fs‐3xMyc plasmids were purchased from Generay Biotech Co., Ltd. Transfection was performed using Lipofectamine 3000.

### 2.5 shRNA‐mediated gene knockdown

For *HECA* knockdown, we used a vector‐based short hairpin RNA (shRNA) expression system. We designed two pairs of nucleotide sequences (Table S2) targeting *HECA*, that were inserted into the pGreen lentiviral shRNA vector. The constructed lentiviral vector carried puromycin resistance, which was used for subsequent screening of stable *HECA*‐shRNA cell lines. HEK293T cells were seeded in a 10‐cm plate and grown to 80% confluence. Then, 12 μg of the constructed pGreen‐*HECA*‐shRNA plasmid and 4.5 μg PMD2 and 7.5 μg of the PSPAX lentivirus packaging plasmid were mixed and added to the 293 T cells according to the Lipo3000 manufacturer's protocol. After culturing for another 48 h, the lentivirus‐containing supernatant was harvested and concentrated according to the manufacturer's protocol. AC16 cells were seeded in 6‐well culture plates and grown to 80% confluence. A concentrated virus solution, which was mixed with culture medium and 10 μg/ml polybrene (Beyotime, China), was added to the AC16 cells. Infected cells were cultured for another 72 h and then screened with 2 μg/ml puromycin (Beyotime, China) for 2 weeks. Next, we verified the knockdown efficiency of the two pairs of shRNA by qPCR and selected the one with better knockdown efficiency for subsequent experiments.

### 2.6 Reverse transcription‐quantitative polymerase chain reaction (RT‐qPCR)

RNA was extracted using TRIZOL reagent (Thermo Fisher Scientific) and reverse‐transcribed into cDNA using the PrimeScript™ RT reagent Kit (Takara). Then, cDNA was amplified by PCR using TB Green® Premix Ex Taq™ (Takara) on a QuantStudio 3 Real‐Time PCR System (Thermo Fisher Scientific), according to the following parameters: denaturation at 95°C for 30 s, PCR reaction of 40 cycles at 95°C for 5 s, 60°C for 30 s, dissociation at 95°C for 5 s, 60°C for 1 min, and 72°C for 45 s. Human *HECA*, *GAPDH*, and other signaling pathway‐related primers are listed in Table S3.

### 2.7 Western blotting

AC16 cells in the pGreen and *HECA*‐shRNA groups were starved with serum‐free medium for 24 h and then stimulated with PDGF‐BB (CST) in time and concentration gradients. For concentration gradient stimulation, AC16 cells were stimulated for 10 min with PDGF‐BB at 0, 10, and 100 ng/ml. For time gradient stimulation, AC16 cells were stimulated with 100 ng/ml PDGF‐BB for 0, 5, and 10 min. AC16 cells were lysed in RIPA buffer containing 100× PMSF (Beyotime, China) and 10× phosphate inhibitor (Beyotime) after stimulation with or without PDGF‐BB. Protein concentrations were determined using the BCA Protein Assay Kit (Takara). Twenty micrograms of protein from each group were loaded and separated using 10% SDS‐polyacrylamide gel (EpiZyme). The proteins were then transferred onto a PVDF membrane (Millipore). The membrane was blocked with 5% BSA at room temperature for 2 h and incubated overnight with the corresponding primary antibody at 4°C. GAPDH (1:1000), PDGF receptor β (1:1000), and phospho‐PDGF receptor β (1:1000) rabbit antibodies were purchased from CST (Danvers). Finally, the membrane was further incubated with the recommended dilution of conjugated secondary antibody at room temperature for 2 h and exposed to peroxide buffer (Tanon). Goat anti‐rabbit IgG and HRP‐linked (1:5000) antibodies were purchased from CST (Danvers).

### 2.8 Cell cycle analysis

AC16 cells were seeded in 6‐well culture plates and grown to 90% confluence. Cells were collected and fixed in 75% ethanol at 4°C overnight. The fixed cells were collected by resuspension and centrifugation. The cells were then stained using the Cell Cycle and Apoptosis Detection Kit (Beyotime) according to the manufacturer's protocol. The data were analyzed using ModFit software.

### 2.9 Cell proliferation test

The proliferation of AC16 cells was assessed using the Cell Counting Kit‐8 (CCK8) (Dojindo, USA). AC16 cells were seeded in 96‐well culture dishes at 4 × 10^3^ cells/well. CCK‐8 (10 μl) solution was added to each well at 0, 24, 48, and 72 h. Then, the AC16 cells were incubated for 4 h and the absorbance at 450 nm was measured after plating the cells.

### 2.10 Scratch assay

AC16 cells were seeded in 6‐well culture dishes. After the AC16 cells were grown to confluence, a scratch was produced using a 20 μl pipette tip (QSP). The cells were washed twice with PBS to remove floating cells and debris. The scratch area was monitored and measured at 0, 12, 24, 36, and 48 h. The data were analyzed using the ImageJ software.

### 2.11 RNA sequencing

RNA sequencing was performed by Jingneng Company.

### 2.12 Statistical analysis

All data are expressed as mean ± SD from at least three independent experiments. All statistical analyses were two‐sided: *p < 0.05, **p < 0.01, and ***p < 0.001. Statistical analyses were performed with GraphPad Prism 8. Student's t test was used to analyze significant differences in the data between the two experimental groups, one‐way ANOVA was used to analyze multiple group comparisons, and Fisher's exact test was used to analyze the association of rare pathogenic *HECA* variants with CHD.

## 3 RESULTS

### 3.1 Identification of *HECA* variants in patients with CHD

**FIGURE 1:**
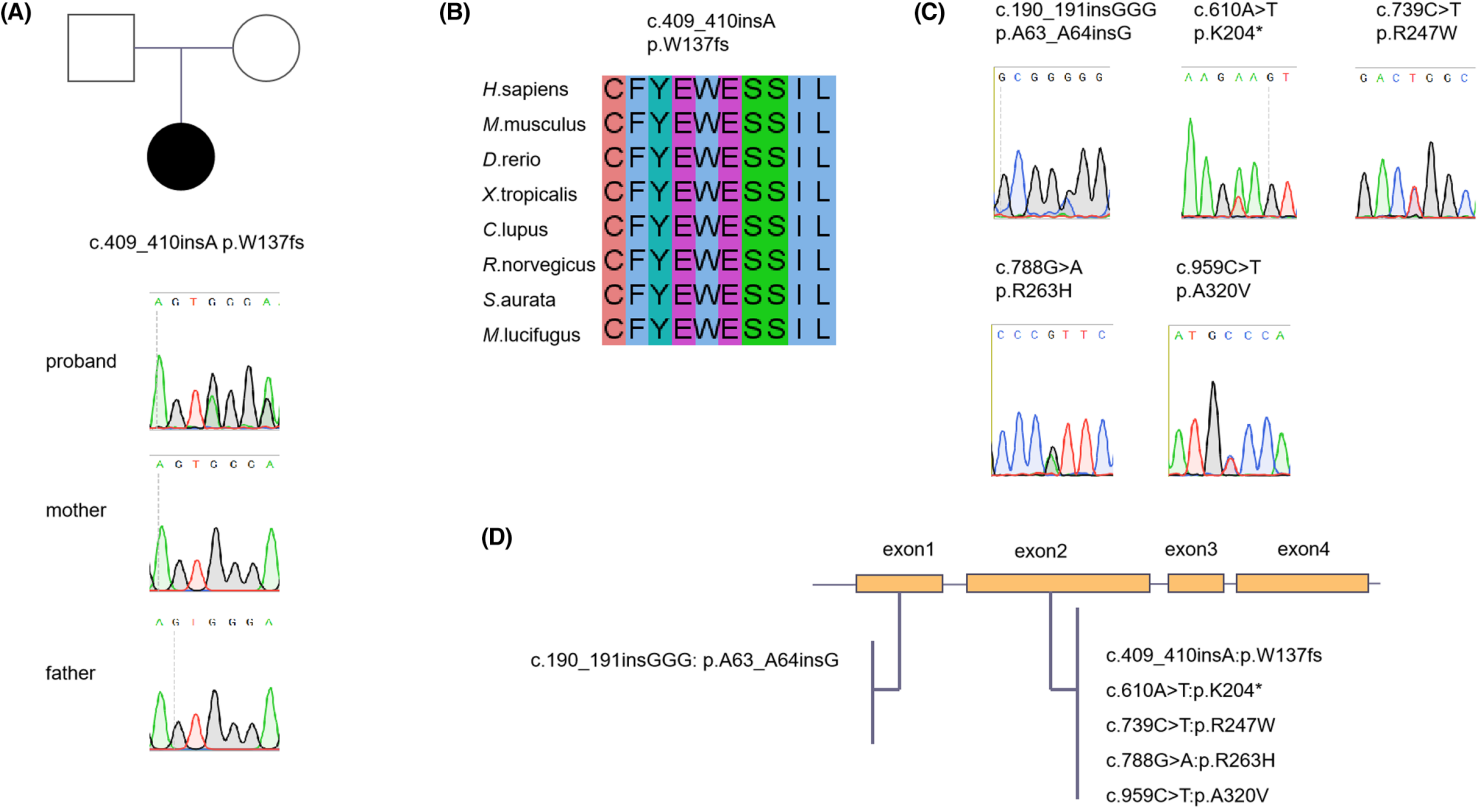
Rare *HECA* variants identified in CHD patients. (A) The family pedigree and Sanger sequencing results. Circle indicates female, square indicates male, and the blackened symbol indicates the proband. (B) Evolutionary conservation of the amino acid W137fs and its surrounding sequences among different species. (C) Sanger sequencing of the other five rare variants identified in sporadic CHD patients. (D) Distribution of rare *HECA* variants in the exons of the gene

We first performed WES in a proband diagnosed with ASD and her healthy parents to explore possible candidate genes. We identified a novel de novo mutation (NM_016217.3, c.409_410insA: p. W137fs) in the *HECA* gene, which was verified by Sanger sequencing. This variant was not present in gnomADv2, suggesting that it was a novel mutation (Table 2). The proband carried W137fs, but her parents did not, indicating that it was a de novo mutation (Figure 1A). The p. W137fs variant is located in exon 2 of the *HECA* gene, whose protein structure is unknown (Figure 1D). The amino acid at position 137 and its surrounding sequence are highly conserved among different species (Figure 1B), indicating that this amino acid is important for the function of *HECA*.

**TABLE 2:** Pathogenicity prediction of rare pathogenic *HECA* variants

Patient ID	Exon	cDNA	Protein	Zygosity	Impact	gnomAD East Asian	CADD	Polyphen2	SIFT	Mutation taster
Wes family										
NO_1570	2	c.409_410insA	p.W137fs	heterozygous	Frameshift variant & stop gained	NA	NA	NA	NA	NA
Targeted sequencing										
NO_2939	1	c.190_191insGGG	p.A63_A64insG	heterozygous	Disruptive in‐frame insertion	NA	NA	NA	NA	NA
NO_1716	2	c.610A > T	p.K204*	heterozygous	Stop gained	NA	NA	40	NA	NA
NO_1856	2	c.739C > T	p.R247W	heterozygous	Missense variant	0.0001	23	B	T	D
NO_0993	2	c.788G > A	p.R263H	heterozygous	Missense variant	0	23.4	D	D	D
NO_0116	2	c.959C > T	p.A320V	heterozygous	Missense variant		23.1	B	T	D

Abbreviations: B, benign; D, disease causing; NA, no data available; T, tolerant.

### 3.2 Association of rare deleterious *HECA* variants with the risk of CHD development

To further investigate the prevalence of damaging *HECA* variants in CHD, we performed targeted exon sequencing of *HECA* in 689 patients with CHD. As shown in Table 2, we observed five novel or rare deleterious heterozygous variants that met the filtering criteria (absence or gnomADv2_exome_EAS frequency ≤0.01% and CADD score > 20). These variants were also verified by Sanger sequencing (Figure. 1C) and were mainly concentrated in exon 2 (Figure 1D). These mutated sites and their surrounding amino acid sequences were well conserved among different species (Figure S1). The clinical information of patients with these variants is shown in Table S4.

To further evaluate the effect of rare deleterious *HECA* variants on CHD risk, we compared the mutation frequency of the *HECA* gene in our study with those in gnomADv2‐East Asians. *HECA* damaging variants were significantly associated with CHD risk (Fisher's exact test, OR: 6.695, 95% CI: 2.543–18.43, *p* = 0.0027), as shown in Table 3.

**TABLE 3:** Comparisons of rare pathogenic variants between sporadic CHD patients and controls

	Allele count (identified *HECA* mutations)	Allele count (no identified *HECA* mutations)	*p*‐value	OR	95% CI lower	95% CI upper
CHD patients	5	1373	0.0027	6.695	2.543	18.43
Controls (gnomAD v2)	10	18,384				

Abbreviation: OR: odds radio.

We also used the Comparative Toxicogenomics Database (CTD) to predict the correlation between *HECA* and congenital malformations. As seen in Table S5, the results showed that *HECA* had certain CHD scores, which further corroborated that *HECA* may play an important role in the occurrence of CHD.

### 3.3 Stable HECA‐deficient AC16 cells

**FIGURE 2:**
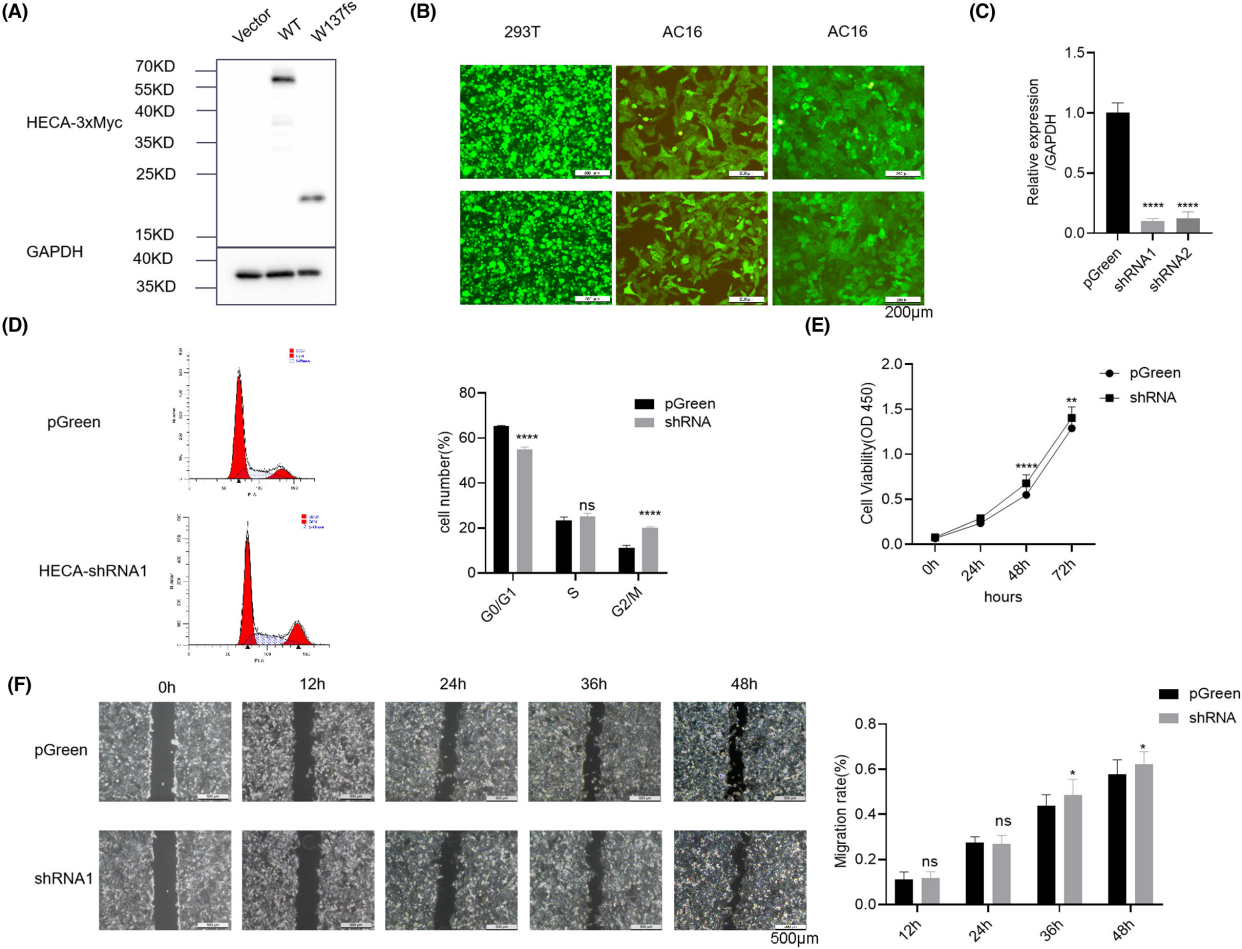
Deficiency of *HECA* in cardiomyocytes alters cell cycle progression and promotes cell proliferation and migration. (A) Western blot analysis of the molecular weight of truncated HECA proteins altered by the W137fs mutation. (B) Construction of *HECA*‐shRNA of AC16 cells. Pictures of HEK293T cells transfected with lentiviral plasmids for 48 h (left, original magnification 100×). Pictures of AC16 cells infected with lentivirus for 72 h (middle, original magnification 100×). Pictures of AC16 cells stably transduced with *HECA*‐shRNA that were screened using puromycin for 2 weeks (right, original magnification 100×). (C) qPCR analysis of the downregulation efficiency of *HECA* in AC16 cells. (D) Representative pictures (left) and quantification (right) of the cell cycle distribution of AC16 cells were detected by flow cytometry analysis. (E) Cell proliferation was analyzed by CCK8 assay. (F) Representative pictures (left, original magnification 40×) and quantification (right) of scratch experiments

To confirm whether the nonsense *HECA* variant p. W137fs could produce a normal HECA protein, we transfected three plasmids, myc‐vector, *HECA*‐wild type, and *HECA*‐p. W137fs with a myc tag into AC16 cells. As shown in Figure 2A, Western blot analysis showed that the p. W137fs variant led to an abnormal protein with a molecular weight less than 20 kDa, which was much lower than that of the wild‐type protein (approximately 70 kDa).

To further address the effect of HECA deficiency on cardiomyocyte function, the expression of *HECA* was downregulated by shRNA‐mediated knockdown in AC16 cells. Three plasmids, including two nucleotide sequences targeting *HECA* (shRNA1 and shRNA2) and an empty control plasmid, were stably transduced into the AC16 cells. As shown in Figure 2B (left panel), when HEK293T cells were transfected with lentiviral plasmids for 48 h, the proportion of cells carrying green fluorescence reached 90%, and the 293 T cells were in good condition. As shown in Figure 2B (middle panel), when AC16 cells were infected with lentivirus for 72 h, the percentage of cells carrying green fluorescence reached 80%, and the AC16 cells were in good condition. As shown in Figure 2B (right panel), when the AC16 cells stably transfected with *HECA*‐shRNA were screened with puromycin for 2 weeks, the number of cells carrying green fluorescence reached 100%, and the AC16 cells were in good condition.

We used qPCR to verify the mRNA level of *HECA*. Compared with the pGreen group, the expression level of *HECA* in the shRNA1 and shRNA groups was significantly reduced (Figure 2C). We selected *HECA*‐shRNA1 AC16 cells, which showed a higher downregulation efficiency of *HECA*, for subsequent experiments.

### 3.4 Effects of HECA deficiency on the cell cycle, proliferation, and migration of AC16 cells

It has been reported that HECA functions in cell cycle and cell proliferation. To determine whether HECA deficiency affects cell cycle regulation in cardiomyocytes, we examined the AC16 cell cycle using flow cytometric analysis. As shown in Figure 2D, HECA deficiency decreased the proportion of AC16 cells in G0/G1 phase and increased the proportion of AC16 cells in G2/GM phase. Downregulation of *HECA* expression in AC16 cells promoted cell proliferation. To further verify the effect of HECA deficiency on cardiomyocyte proliferation, we performed CCK8 experiments. There was no significant difference in cell proliferation between the two groups at 24 h, while the proliferation ability of AC16 cells in the *HECA*‐shRNA group was much higher than that of the control group at 48 and 72 h (Figure 2E).

To assess the effect of *HECA* deficiency on cardiomyocytes migrations, a scratch assay was performed on AC16 cells. As shown in Figure 2F, there was no obvious change in the migration ability of AC16 cells at 12 and 24 h, but downregulation of *HECA* expression significantly increased the migration ability of AC16 cells at 36 and 48 h. These data indicate that HECA deficiency in cardiomyocytes alters cell cycle progression and promotes cell proliferation and migration.

### 3.5 Effects of HECA deficiency on downstream signaling pathways in cardiomyocytes

**FIGURE 3:**
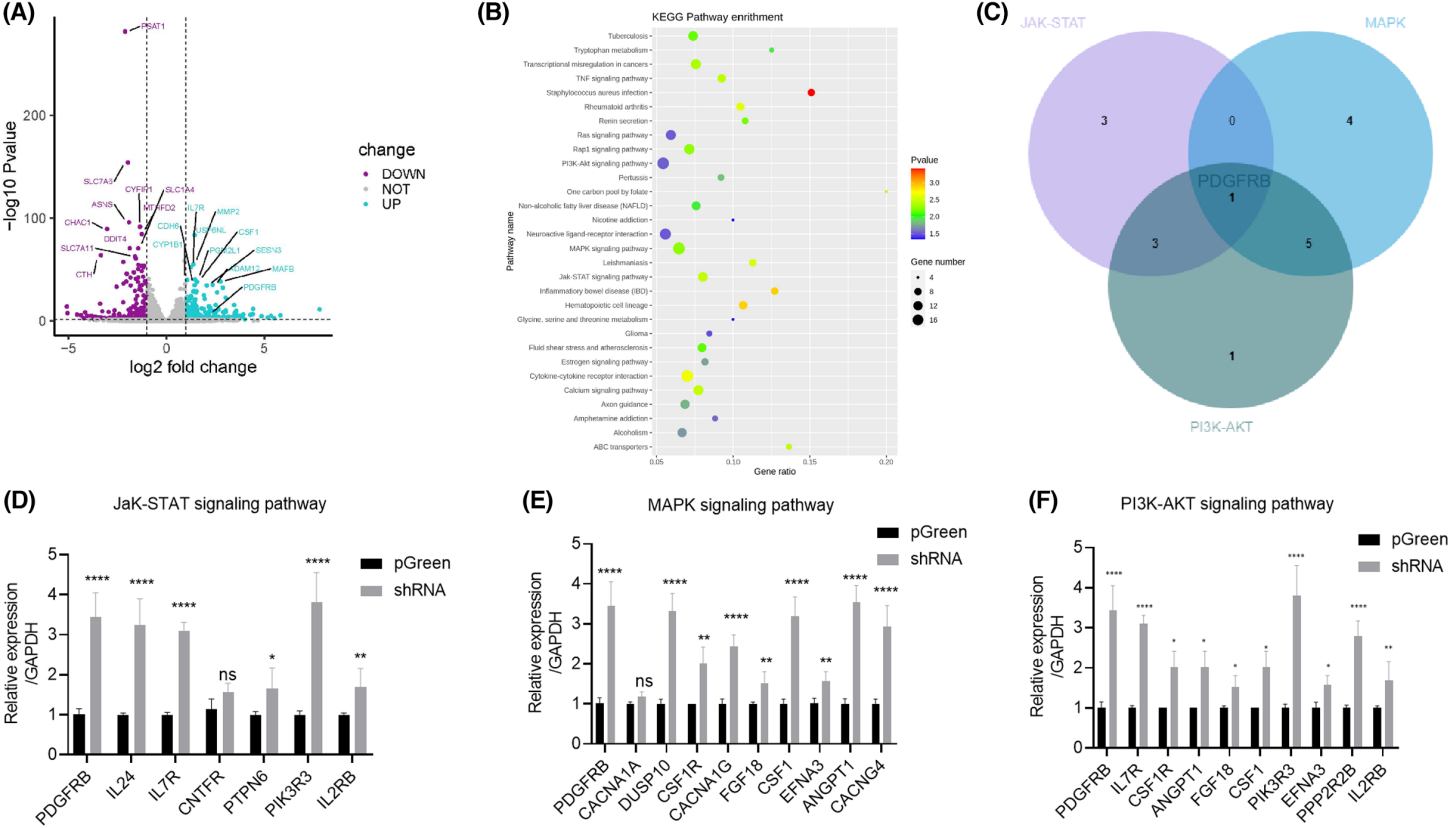
Effects of *HECA* deficiency on downstream signaling pathways in cardiomyocytes. (A) Volcano plot of differentially expressed genes after *HECA* knockdown. (B) Representative gene ontology term analysis of differentially expressed genes after *HECA* knockdown. (C) The JAK–STAT, MAPK, and PI3K‐AKT pathways showed an overlap of one gene, *PDGFRB* after *HECA* knockdown. (D) Differentially expressed genes in the JAK–STAT pathway, (E) the MAPK pathway, and (F) the PI3K‐AKT pathway were detected by qPCR.

To understand the mechanism by which HECA regulates AC16 cell proliferation and migration, we used mRNA sequencing to explore the process of *HECA* regulation. Compared with the pGreen group, the *HECA* knockdown group had 1185 differentially expressed genes that met the criteria of |log2(fold change) > 1| and *p* ≤ 0.05, of which 704 genes were upregulated and 481 genes were downregulated (Figure 3A). As shown in Figure 3B, downregulation of *HECA* in AC16 cells significantly affected multiple signaling pathways, including TNF, JAK–STAT, cytokine interaction, MAPK, axon guidance, PI3K‐Akt, glioma, and other signaling pathways, among which JAK–STAT, MAPK, and PI3K‐Akt are closely related to cell proliferation and migration. By analyzing the RNA sequencing data, we found that PDGFRB was significantly upregulated in all three pathways (Figure 3C).

We then used qPCR to verify the related representative genes of the JAK–STAT, MAPK, and PI3K‐KAT pathways obtained by mRNA sequencing and found that *PDGFRB*, *IL24*, *IL7R*, *PTPN6*, *PIK3R3*, and *IL2RB* were significantly upregulated in the JAK–STAT signaling pathway (Figure 3D). *PDGFRB, DUSP10*, *CSF1R*, *CACNA1G*, *FGF18*, *CSF1*, *EFNA3*, *ANGPT1*, and *CACNG4* were significantly upregulated in the MAPK signaling pathway (Figure 3E). *PDGFRB*, *IL7R*, *CSF1R*, *ANGPT1*, *FGF18*, *CSF1*, *PIK3R3*, *EFNA3*, *PPP2R2B*, and *IL2RB* were significantly upregulated in the PI3K‐AKT signaling pathway (Figure 3F).

### 3.6 HECA deficiency promotes PDGF‐BB‐induced activation of PDGFRB and downstream Akt

**FIGURE 4:**
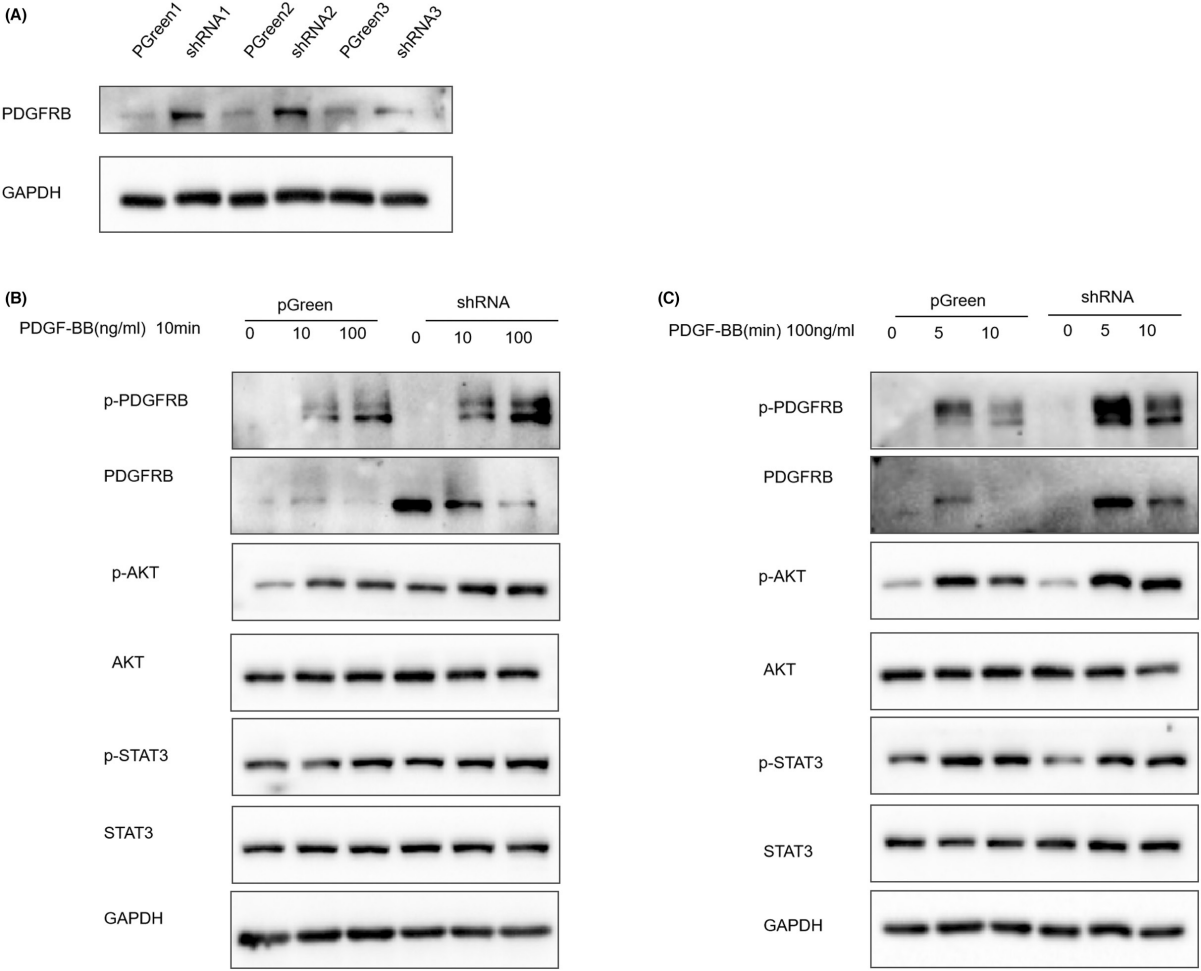
Deficiency of *HECA* in cardiomyocytes promotes activation of the PDGF‐BB/PDGFRB/AKT pathway. (A) Representative pictures (left) and quantification (right) of PDGFRB expression in the pGreen and *HECA*‐shRNA groups were detected by western blot. (B) The relative protein expression of p‐PDGFRB, p‐AKT, and p‐STAT3 was detected by western blot after treatment with 0 ng/ml, 10 ng/ml, and 100 ng/ml PDGF‐BB for 10 min. (C) The relative protein expression of p‐PDGFRB, p‐AKT, and p‐STAT3 was detected by western blot at 0, 5, and 10 min of treatment with 100 ng/ml PDGF‐BB.

By analyzing the qPCR validation results, we found that the *PDGFRB* gene was significantly upregulated in all three pathways. Western blotting was performed to further investigate the effect of *HECA* deficiency on PDGFRB expression. The results showed that PDGFRB protein expression was significantly increased after *HECA* downregulation in AC16 cells (Figure 4A).

We subsequently explored whether *HECA* deficiency in AC16 cells affects ligand‐dependent activation of PDGFRB and its downstream AKT and STAT3 signaling. Western blotting was performed after AC16 cells were starved for 24 h and stimulated with PDGF‐BB at different concentrations and time gradients. As shown in Figure 4B, p‐PDGFRB was significantly upregulated in the HECA‐shRNA group compared with the control group under 100 ng/ml treatment. The change in p‐AKT was consistent with the changes in p‐PDGFRB, but p‐STAT3 did not change significantly. As depicted in Figure 4C, p‐PDGFRB levels were significantly greater in the HECA‐shRNA group after 5 min of stimulation. Similarly, the alteration in p‐AKT levels was consistent with that in p‐PDGFRB. However, there was no significant alteration in p‐STAT3.

These results indicated that HECA deficiency in cardiomyocytes promoted the PDGF‐BB/PDGFRB/AKT pathway.

## 4 DISCUSSION

Cardiac development is a complex and rigorously dynamic process, and cardiomyocyte proliferation plays an important role in early cardiac development. Impaired regulatory mechanisms of cardiomyocyte proliferation and cycle progression can lead to abnormal cardiac chamber formation and ventricular remodeling.^23^ *HECA* has rarely been reported to be a cell cycle regulator in CHD.

In this study, we performed whole‐exome sequencing on a family with ASD and found a novel, de novo, and pathogenic *HECA* mutation (c.409_410insA (p. W137fs)) in a proband. To assess the association of *HECA* deleterious variants with CHD, we performed targeted exon sequencing of the *HECA* gene in additional 689 patients with sporadic CHD. When compared to the East Asian population in GnomADv2, we found a significant association between *HECA* variations and CHD (*p* = 0.0027) and an odds ratio of 6.695 for CHD in patients with *HECA*. Therefore, it is worth considering to include *HECA* in the polygenic panel of Han patients with CHD. The *HECA* gene is composed of four exons, and its protein domains remain unclear. The six rare and deleterious variants detected in this study were mainly concentrated in exon 2, suggesting that the second exon of *HECA* may be important for cardiovascular development and CHD occurrence.

Cardiovascular development can be divided into two important growth stages: early embryonic development and fetal cardiac growth. Early embryonic development mainly depends on the extensive proliferation of progenitor cells in the second heart field, and these progenitors are gradually added to the outflow tract (OFT) cardiac neural crest cells migrate from the dorsal neural tube to the OFT, whereas fetal heart growth is dependent on the proliferation of differentiated cardiomyocytes.^10^, ^24^ Abnormal proliferation and migration of cardiomyocytes can affect normal cardiac development and lead to CHD. Truncated proteins produced by frameshift mutations may lead to gene loss‐of‐function due to haploinsufficiency.^25^, ^26^, ^27^ *HECA* has a modest probability of a loss‐of‐function intolerance score (0.615) and a loss‐of‐function observed/expected upper bound fraction score (0.48).^28^ The nonsense *HECA* variant p. W137fs is caused by a single nucleotide (A) insertion, resulting in premature termination of translation after 137 codons. We determined by Western blotting that the variant p. W137fs could lead to a deficiency in the normal HECA protein. We found that HECA deficiency in AC16 cells decreased the proportion of AC16 cells in the G0/G1 phase and promoted their proliferation. This result was in line with previous reports in which silenced *HECA* promoted tumor cell proliferation in tumor cells.^29^ We also observed that HECA deficiency in AC16 cells promoted cell migration, which differed from findings in tumor cells. Wang et al.^29^ found that neither overexpression nor downregulation of *HECA* affected the migration ability of HepG2, Huh‐7, and MHCC‐97H cells; however, they only detected cell migration at 24 h. Our scratch experiments showed that knockdown of *HECA* did not affect cell migration ability at 12 and 24 h, but at 36 and 48 h it significantly promoted migration. Therefore, we considered that the varying effects of *HECA* on migration ability might be due to the fact that cell migration ability was not assessed after 24 h in the previous study.

In our study, we observed that HECA deficiency in AC16 cells increased PDGFRB expression. PDGFRB, platelet‐derived growth factor‐B, belongs to the PDGF family, which plays a critical role in cardiovascular development.^30^ Previous studies have shown that after the deletion or blockade of PDGF and its receptor, PDGFR, in mouse models, mouse embryos typically die in the early fetal or neonatal period and exhibit severe cardiac malformations.^31^ PDGFRA, a member of the PDGFRB family, is associated with the development of total anomalous pulmonary venous return syndrome, a serious type of CHD caused by ASD or pulmonary veins not draining into the left atrium.^32^ Noortje et al.^33^ reported that *Pdgfra*‐deficient mouse embryos displayed cardiac malformation phenotypes, including atrial and venous sinus myocardial hypoplasia. PDGF‐B/PDGFRβ signaling is critical for myocardial development, coronary vascular development, and cardiac nerves, and PDGF‐B and PDGFRB‐knockout mouse embryos developed cardiac phenotypes such as myocardial hypoplasia, coronary dilatation, and atrioventricular valve dysplasia.^34^ Previous reports have indicated that the PDGFR inhibitor AG1296 inhibits PDGF‐BB‐induced myoblast cell cycle progression, proliferation, migration, and AKT activation.^35^ Wang et al.^36^ discovered that PDGF‐BB regulates PDGFRB/PI3K/AKT pathway activity to induce the proliferation and migration of oral mucosal fibroblasts. We observed that deficiency of *HECA* in AC16 cells increased PDGF‐BB/PDGFRB/AKT pathway activity. Therefore, we hypothesized that the *HECA* deficiency in AC16 cells promotes cell proliferation and migration by modulating the activity of the PDGF‐BB/PDGFRB/AKT pathway, which in turn may affect cardiac development.

Our study is the first to report the frequency of rare variants of *HECA* in CHD and to analyze the association between *HECA* variants and the risk of CHD, which expands the polygenic panel of Han patients with CHD and provides new insights for the screening and prevention of CHD. However, some limitations require further exploration. First, we were unable to obtain cardiac tissue from CHD patients to detect normal HECA protein levels and abnormal truncating mutations. Second, our study mainly focused on an AC16 cell model. Therefore, further studies on the specific role and pathogenic mechanism of the *HECA* gene and its mutants in the occurrence of CHD are warranted.

## 5 CONCLUSIONS

We reported six rare deleterious variants in the *HECA* gene from a family with ASD and 689 patients with sporadic CHD. Our findings revealed that *HECA* damaging variants are associated with CHD risk, which expands the spectrum of genes associated with the pathogenesis of CHD in the Chinese population. These functional results indicate that HECA may affect the proliferation and migration of cardiomyocytes by modulating the PDGF‐BB/PDGFRB/AKT signaling pathway, thereby affecting cardiac development.

## AUTHOR CONTRIBUTIONS

TL, YW, and WC collected patient information, performed the experiments, analyzed the data, and wrote the original study. XX, MS, and PL helped with the cell culture, cell passage, and data analysis. WS and GH designed the study and edited and revised the final draft. All authors have read and approved the final study.

## FUNDING INFORMATION

This work was supported by the National Key Research and Development Program of China (Grant No. 2021YFC2701000, 2016YFC1000500), National Natural Science Foundation of China (Grant No. 81873482, 81873483, 81800282), Shanghai Basic Research Project of Science and Technology Innovation Action Plan (Grant Number: 20JC1418300), and CAMS Innovation Fund for Medical Sciences (2019‐I2M‐5‐002).

## CONFLICT OF INTEREST

The authors declare that they have no competing interests.

## Supporting information


Appendix S1

## Data Availability

All data relevant to the study are presented in the paper, and all the results contain raw data.
