# Supplementary material for: Functional analysis of HECA variants identified in congenital heart disease in the Chinese population
Source: J Clin Lab Anal. 2022 Aug 10;36(9):e24649. doi: 10.1002/jcla.24649 (PMC9459261; doi:10.1002/jcla.24649)
Supplement: Supplementary file 1 — Appendix S1 [file JCLA-36-e24649-s001.docx]

**Supplementary Figures and Tables**


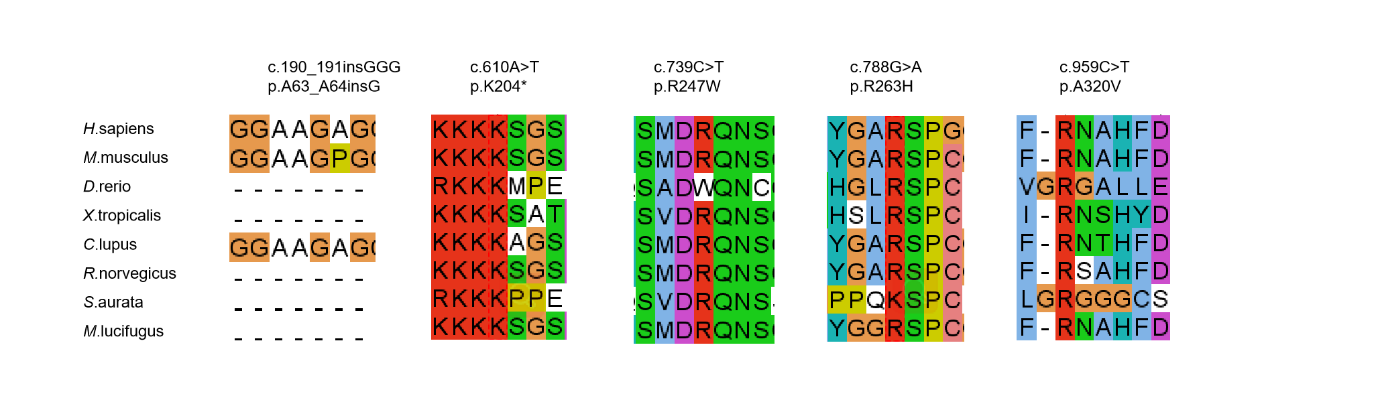


**Supplementary Figure 1** Conservation of rare variants in the *HECA* gene found in patients with sporadic CHD.

**Supplementary Table 1** Sanger sequencing primer pair for detecting *HECA* variants.

| Primer name | Sequences 5′-3′ |
| --- | --- |
| Exon1-F | ATGCCCAACCCCAAAAACAGC |
| Exon1-R | CGTTTTTGGCATCGCCCGC |
| Exon2-F | AAGCCCCATGTGCCACTCC |
| Exon2-R | CTTGAAGGTGACAAGGAACATCA |

F: forward primer, R: reverse primer.

**Supplementary Table 2** Primer pairs for *HECA* shRNA.

| **Primer name** | **Sequences 5′-3′** |
| --- | --- |
| *HECA*-shRNA1-sense | GATCCCGAGACATGATGAGATCGAATCTCG  AGATTCGATCTCATCATGTCTCGTTTTTTG |
| *HECA*-shRNA1-antisense | AATTCAAAAAACGAGACATGATGAGATCGA  ATCTCGAGATTCGATCTCATCATGTCTCGG |
| *HECA*-shRNA2-sense | GATCCGAAGACTTGCGGAAGTTCATTCTCG  AGAATGAACTTCCGCAAGTCTTCTTTTTTG |
| *HECA*-shRNA2-antisense | AATTCAAAAAAGAAGACTTGCGGAAGTTCA  TTCTCGAGAATGAACTTCCGCAAGTCTTCG |

**Supplementary Table 3** Primer pairs for real-time qPCR analysis.

| **Primer name** | **Sequences 5′-3′** |
| --- | --- |
| human-*GAPDH*-F | GGAGCGAGATCCCTCCAAAAT |
| human-*GAPDH*-R | GGCTGTTGTCATACTTCTCATGG |
| human-*HECA*-F | AACGTGGTAAACTGTGCCCT |
| human-*HECA*-R | ACAGATGCATGAGTCTCCCTTG |
| human-*PDGFRB*-F | TGATGCCGAGGAACTATTCATCT |
| human-*PDGFRB*-R | TTTCTTCTCGTGCAGTGTCAC |
| human-*CACNA1A*-F | CGCTTCGGAGACGAGATGC |
| human-*CACNA1A*-R | TGCGCCATTGACTGCTTGT |
| human-*IL24*-F | CACACAGGCGGTTTCTGCTAT |
| human-*IL24*-R | TCCAACTGTTTGAATGCTCTCC |
| human-*CNTFR*-F | CTGGGCTCTGACGTGACAC |
| human-*CNTFR*-R | GTGGAAGCAGGCGTAGAGG |
| human-*IL7R*-F | CCCTCGTGGAGGTAAAGTGC |
| human-*IL7R*-R | CCTTCCCGATAGACGACACTC |
| human-*PTPN6*-F | GGTGTCCACGGTAGCTTCC |
| human-*PTPN6*-R | ACAGGTCATAGAAATCCCCTGAG |
| human-*PIK3R3*-F | TACAATACGGTGTGGAGTATGGA |
| human-*PIK3R3*-R | TCATTGGCTTAGGTGGCTTTG |
| human-*IL2RB*-F | CAGCGGTGAATGGCACTTC |
| human-*IL2RB*-R | GGCATGGACTTGGCAGGAA |
| human-*CACNA1G*-F | TGTCTCCGCACGGTCTGTAA |
| human-*CACNA1G*-R | AAGCCGGTTCCAAGTGTCTC |
| human-*CSF1*-F | TGGCGAGCAGGAGTATCAC |
| human-*CSF1*-R | AGGTCTCCATCTGACTGTCAAT |
| human-*PPP2R2B*-F | CCACACGGGAGAATTACTAGCG |
| human-*PPP2R2B*-R | TGTATTCACCCCTACGATGAACC |
| human-*DUSP10*-F | ATCGGCTACGTCATCAACGTC |
| human-*DUSP10*-R | TCATCCGAGTGTGCTTCATCA |
| human-*CSF1R*-F | GGGAATCCCAGTGATAGAGCC |
| human-*CSF1R*-R | TTGGAAGGTAGCGTTGTTGGT |
| human-*ANGPT1*-F | AGCGCCGAAGTCCAGAAAAC |
| human-*ANGPT1*-R | TACTCTCACGACAGTTGCCAT |
| human-*CACNG4*-F | CATCGAAGGGATCTATAAAGGGC |
| human-*CACNG4*-R | GAGGAGGTACTCCGAGCTGT |
| human-*FGF18*-F | ACTTGCCTGTGTTTACACTTCC |
| human-*FGF18*-R | GACCTGGATGTGTTTCCCACT |
| human-*EFNA3*-F | CATGCGGTGTACTGGAACAG |
| human-*EFNA3*-R | AGATAGTCGTTCACGTTCACCT |

F: forward, R: reverse.

**Supplementary Table 4** Clinical information of CHD patients with rare pathogenic *HECA* variants.

| **Patient ID** | **Gender** | **Age(months)** | **Phenotype** | |
| --- | --- | --- | --- | --- |
|  |  |  | **CHD** | **Extracardiac malformations** |
| **WES family** |  |  |  |  |
| NO_1570 | F | 10.93 | ASD | Mental retardation, Face abnormal |
| **Targeted sequencing** |  |  |  |  |
| NO_2939 | F | 12.13 | ASD | Hypothyroidism, Growth retardation |
| NO_1716 | F | 7.90 | VSD | Inguinal hernia |
| NO_1856 | M | 0.93 | TGA |  |
| NO_0993 | M | 0.53 | TGA |  |
| NO_0116 | M | 44.63 | SV | VUR |

ASD, Atrial septal defect, VSD, Ventricular septal defect, TGA, Transposition of the great arteries, SV: single ventricle, VUR: vesicoureteral reflux.

**Supplementary Table 5** Correlation scores between *HECA* and congenital malformations and congenital heart disease in the CTD database.

| **Gene symbol** | **Disease** | **Direct evidence** | **Inference Score** |
| --- | --- | --- | --- |
| *HECA* | Congenital Abnormalities | 0 | 61.20 |
|  | Heart Defects, Congenital | 0 | 47.19 |
|  | Heart Septal Defects, Ventricular | 0 | 21.20 |
|  | Heart Septal Defects, Atrial | 0 | 12.84 |
